# Oral Conditions, Salivary pH, Flow Rate, Phosphate Level, and Phosphorus Intake of Pre- and Postmenopausal Women

**DOI:** 10.1055/s-0044-1795076

**Published:** 2024-12-10

**Authors:** Sri Tjahajawati, Anggun Rafisa, Namira Vahra Khairunnisa Aldrin, Hening Tjaturina Pramesti

**Affiliations:** 1 Department of Oral Biology, Faculty of Dentistry, Universitas Padjadjaran, Indonesia; 2 Bachelor of Dentistry Study Program, Faculty of Dentistry, Universitas Padjadjaran, Bandung, Indonesia

**Keywords:** intraoral conditions, saliva, pH, flow rate, phosphate level, phosphorus intake, menopause

## Abstract

**Objectives:**

This study aimed to investigate salivary parameters, including pH, flow rate, phosphate levels, and phosphorus intake, to explore potential variations between postmenopausal and premenopausal women.

**Material and Methods:**

This study included 68 postmenopausal and 94 premenopausal women. Inclusion criteria comprised women aged 45 to 65 years with a minimum menopausal duration of 2 years and women aged 21 to 40 years for premenopausal participants. Exclusion criteria involved systemic diseases and any dental treatments received in the last 6 months. Direct observation facilitated the visual detection of intraoral inflammation, ulcers, plaque, calculus, dental mobility, and caries. A questionnaire covering demographic data, dental pain, xerostomia, burning sensation, ulcer etiology and duration, and gingival bleeding etiology was administered. Phosphorus intake was assessed using a semiquantitative food frequency questionnaire. Unstimulated whole saliva, collected by spitting, was analyzed for various salivary parameters, such as pH, flow rate, and phosphate level. The atomic absorption spectrophotometer was used to determine subjects' salivary phosphate level.

**Statistical Analysis:**

The difference in intraoral conditions between groups was analyzed using the chi-square or Fisher's exact test. For the comparison of salivary parameters and phosphorus intake between study groups, the ANOVA: univariate general linear model was utilized. The correlation between salivary phosphate levels and age, body mass index (BMI), blood pressure, and phosphorus intake was examined using Spearman's rank correlation.

**Results:**

The postmenopausal group demonstrated a significantly higher prevalence ( *p* < 0.005) of gingival swelling, gingival discoloration, gingival recession, plaque, calculus, caries, tooth mobility, xerostomia, and burning sensation. Following adjustments for age, BMI, and blood pressure, a statistically significant difference in salivary flow rate between groups was observed ( *p* = 0.008). No significant differences were found in salivary pH ( *p* = 0.764), salivary phosphate level ( *p* = 0.142), or phosphorus intake ( *p* = 0.323) between the two groups. There was no significant correlation between salivary phosphate levels and age ( *p* = 0.747), BMI ( *p* = 0.308), systolic blood pressure ( *p* = 0.747), diastolic blood pressure ( *p* = 0.622), and phosphorus intake (0.829) in both groups.

**Conclusion:**

Postmenopausal women exhibited a lower salivary flow rate compared with premenopausal women. No differences or correlations were observed in salivary phosphate level and phosphorus intake between the two groups.

## Introduction

Menopause, a natural physiological process that typically occurs between the ages of 49 to 52, marks the permanent cessation of the menstrual cycle for 12 consecutive months. 1 Menopause involves hormonal fluctuations, particularly a decline in estrogen levels, which affect diverse bodily systems, including the oral cavity. 2 Despite numerous investigations into the implications of menopause on oral health parameters, findings remain inconsistent. While some studies assert an increased vulnerability to periodontal diseases among postmenopausal women, 2 3 4 others report no significant difference in periodontal disease severity between premenopausal and postmenopausal cohorts, suggesting influences beyond menopause play a pivotal role. 5 Xerostomia and burning mouth syndrome emerge as common menopausal symptoms in certain studies, 2 6 7 8 contrasting with findings that report no such symptoms in postmenopausal women. 9 Additionally, conflicting results surround salivary flow rates, with some studies indicating lower rates in postmenopausal women compared with their premenopausal counterparts, 8 10 11 while others find no significant distinctions. 12 Salivary pH levels in postmenopausal women also elicit divergent findings, with some studies reporting a statistically significant decrease, 10 13 others indicating no change, 14 and some revealing a statistically significant increase. 15

The impact of menopause on salivary flow rate can potentially lead to changes in salivary components, including phosphate. Salivary phosphate, in conjunction with other elements such as calcium, bicarbonate, and proteins, plays a crucial role in regulating saliva's buffering capacity and influencing the processes of dental demineralization and remineralization. 16 The intake of phosphorus further contributes to intraoral conditions. Previous studies have identified that an excessive intake of phosphorus is associated with the development of gingivitis and caries. 17 18 In postmenopausal women, research indicates higher salivary phosphate levels compared with premenopausal women. 19 20 Furthermore, postmenopausal women with diabetes mellitus exhibit higher levels of both stimulated and unstimulated salivary phosphate compared with their nondiabetic counterparts. 21 However, to our knowledge, no studies have comprehensively compared salivary phosphate levels and phosphorus intake, nor have examined their correlation between postmenopausal and premenopausal women.

This study aimed to investigate salivary parameters, pH, flow rate, and phosphate levels, along with phosphorus intake, to identify potential variations between postmenopausal and premenopausal women. Furthermore, it seeks to explore the correlation between salivary phosphate levels and phosphorus intake in both groups. Such insights are essential for comprehending the unique oral health challenges associated with menopause, contributing to a broader understanding of women's health and well-being.

## Material and Methods

Employing purposive sampling, this cross-sectional study included 68 postmenopausal and 94 premenopausal women, conducted in Bandung, West Java, Indonesia, from January to April 2022. Inclusion criteria comprised women aged 45 to 65 years with a minimum menopausal duration of 2 years and women aged 21 to 40 years for premenopausal participants. All subjects were asked to sign the informed consent prior to data collection. All subjects provided informed consent, and only those who completed all the research procedures were included. Exclusion criteria involved systemic diseases (diabetes mellitus, kidney disease, or hyperparathyroidism) and any dental treatments (scaling, orthodontic treatment, denture, or dental restoration) received in the last 6 months.

The assessed variables included physical status (weight, height, and blood pressure), intraoral conditions (inflammation signs, lesions, and disorders), salivary parameters, and phosphorus intake. Intraoral inflammation, such as gingival bleeding, discoloration, swelling, and recession, along with ulcers, plaque, calculus, tooth mobility, and caries, was detected through direct observation using a dental mirror. The examination results only recorded the presence or absence of these intraoral conditions. A questionnaire covering demographic data, dental pain, xerostomia, burning sensation, ulcer etiology and duration, and gingival bleeding etiology was administered. Phosphorus intake was assessed using a semiquantitative food frequency questionnaire consisting of 37 food items with pictures and portion sizes. The questionnaire's validity and reliability were tested on 20 subjects from each postmenopausal and premenopausal group.

Unstimulated whole saliva was collected using the spitting method. The salivary flow rate was calculated by measuring the total volume of saliva collected over the 5-minute period. Salivary pH was determined using a universal indicator pH paper (pH 0–14 Universal Indicator pH Paper, Merck, Darmstadt, Germany). The Atomic Absorption Spectrophotometer AAnalyst 400 (PerkinElmer, Waltham, MA, USA) was used to determine subjects' salivary phosphate level.

### Statistical Analysis

The analysis of the significance difference in intraoral conditions between groups utilized the chi-square test. However, in cases where expected counts in cells were less than five, the Fisher's exact test was employed. For the comparison of salivary parameters and phosphorus intake between study groups, adjusted by age, body mass index (BMI), and blood pressure, the ANOVA univariate general linear model was utilized. The correlation between salivary phosphate levels and age, BMI, blood pressure, and phosphorus intake was examined using Spearman's rank correlation. Statistical significance was defined as *p* < 0.05. All analyses were conducted using SPSS (IBM Corp, Version 26.0, Armonk, NY).

## Results

As illustrated in Table 1 , the mean age of postmenopausal group was 53.4 (standard deviation: 6.6). The majority of subjects in this group (76.5%) had been menopausal for more than 12 months. The postmenopausal group also exhibited higher BMI and blood pressure (both systolic and diastolic) compared with the premenopausal group. Table 2 reveals a statistically significant difference in the proportion of subjects experiencing 10 out of the 14 assessed intraoral conditions between the two groups. The postmenopausal group demonstrated a significantly higher prevalence ( *p* < 0.005) of gingival swelling, gingival discoloration, gingival recession, plaque, calculus, caries, tooth mobility, xerostomia, and burning sensation.

**Table 1:** Characteristics of study subjects

Characteristic	Postmenopausal group		Premenopausal group	
	( *n* = 68)		( *n* = 94)	
	Mean (SD)	Median (range)	Mean (SD)	Median (range)
Age (y)	53.4 (6.6)	54 (21–75)	26.7 (8.5)	21 (19–49)
BMI (kg/m ^2^ )	26.9 (4.4)	27.3 (17.1–38.0)	23.2 (5.4)	21.9 (16.0–38.0)
Systolic blood pressure (mmHg)	139.4 (24.0)	132 (97–201)	115.7 (15.4)	115 (90–156)
Diastolic blood pressure (mmHg)	86.3 (11.6)	83 (63–116)	81.5 (10.7)	80 (64–111)

Abbreviation: SD, standard deviation.

**Table 2:** Intraoral conditions of study subjects

Variables	Postmenopausal group ( *n* = 68)		Premenopausal group ( *n* = 94)		*p* -Value
	Count	Percentage (%)	Count	Percentage (%)	
Gingival swelling	18	26.5	12	12.8	0.027 a
Gingival bleeding (nonspontaneous)	13	19.1	33	35.1	0.026 a
Gingival bleeding (spontaneous)	5	7.4	5	5.3	0.743
Gingival discoloration	21	30.9	16	17.0	0.038 a
Gingival recession	44	64.7	20	21.3	<0.001 a
Ulcers (nontraumatic)	10	14.7	16	17.0	0.692
Ulcers (traumatic)	14	20.6	12	12.8	0.181
Plaque	52	76.5	58	61.7	0.047 a
Calculus	49	72.1	41	43.6	<0.001 a
Caries	63	92.6	64	68.1	<0.001 a
Tooth mobility	27	39.7	10	10.6	<0.001 a
Dental pain	30	44.1	41	43.6	0.949
Xerostomia	21	30.9	16	17.0	0.038 a
Burning sensation	9	13.2	4	4.3	0.038 a

a Statistically signiﬁcant ( *p* -value < 0.05) according to chi-square test or Fisher's exact test.

In Table 3 , following adjustments for age, BMI, and blood pressure, a statistically significant difference in salivary flow rate between groups was observed ( *p* = 0.008), with the postmenopausal group exhibiting lower mean and median salivary flow rate. However, no significant differences were found in salivary pH ( *p* = 0.764), salivary phosphate level ( *p* = 0.142), or phosphorus intake ( *p* = 0.323) between the two groups. As indicated in Table 4 , there was no significant correlation between salivary phosphate levels and subject characteristics, including age ( *p* = 0.747), BMI ( *p* = 0.308), systolic blood pressure ( *p* = 0.747), diastolic blood pressure ( *p* = 0.622), and phosphorus intake (0.829) in both groups.

**Table 3:** Comparison of salivary parameters and phosphorus intake between study groups adjusted for age, BMI, and blood pressure

Variables	Postmenopausal group ( *n* = 68)		Premenopausal group ( *n* = 94)		*p* -Value
	Mean (SD)	Median (range)	Mean (SD)	Median (range)	
Salivary pH	6.07 (0.47)	6 (5–7)	6.21 (0.48)	6 (5–7)	0.764
Salivary flow rate (mL/5 minutes)	1.90 (0.66)	2 (1–4)	3.05 (1.40)	3 (1–8.5)	0.008 a
Salivary phosphate level (mmol/L)	23.17 (11.14)	21.63 (2.58–52.31)	14.53 (5.79)	13.56 (4.49–28.81)	0.142
Phosphorus intake (mg/d)	1103.8 (766.6)	956.7 (398.0–5424.3)	1179.5 (905.0)	939.6 (387.3–5693.4)	0.323

Abbreviation: SD, standard deviation.

a Statistically signiﬁcant ( *p* -value < 0.05) according to ANOVA: univariate general linear model.

**Table 4:** Correlation between salivary phosphate levels with subject characteristics and phosphorus intake

The correlation of salivary phosphate level with	Postmenopausal group ( *n* = 68)		Premenopausal group ( *n* = 94)	
	*r*	*p* -Value	*r*	*p* -Value
Age (y)	0.147	0.337	0.057	0.747
BMI (kg/m ^2^ )	0.194	0.202	0.177	0.308
Systolic blood pressure (mmHg)	0.069	0.650	0.057	0.747
Diastolic blood pressure (mmHg)	−0.009	0.953	0.086	0.622
Phosphorus intake (mg/daily)	0.006	0.968	–0.038	0.829

Note: *r* is Spearman's rank coefficient correlation.

## Discussion

### Subject Characteristics

The observed higher BMI in the postmenopausal group compared with the premenopausal group in this study aligns with findings from previous studies. 22 23 The changes in fat distribution, loss of lean body mass, and the overall increase in body weight and composition that occur with menopause are associated with an elevated risk of obesity in postmenopausal women. 24 Furthermore, Chen et al reported that postmenopausal women experiencing moderate/high levels of stress, inadequate sleep (<6 h/d), and not breastfeeding their infants were at an increased risk of obesity. 22 25 This should draw more attention, as obesity is linked to a wide range of comorbidities and an increased risk of cardiovascular-associated and cancer-associated mortality. 26 Additionally, obese postmenopausal women are more likely to experience hot flushes, sweating, and feeling bloated in the perimenopausal period and increased vasomotor syndrome in the postmenopausal period compared with normal-weight women. 27 The higher BMI observed in postmenopausal women in this study may also be attributed to higher blood pressure, in addition to the decrease in estrogen levels. 28 29

### Intraoral Conditions

This study reveals a significantly higher proportion of postmenopausal women who experienced gingivitis symptoms (gingival swelling, bleeding, and discoloration) and periodontitis indicators (gingival recession and tooth mobility) compared with premenopausal counterparts. Consistent with previous research, women exhibit an increased susceptibility to periodontal diseases during the postmenopausal period. 2 3 4 The alterations in periodontal tissue due to estrogen deficiency and advancing age in postmenopausal women contribute to these findings. 2 3 30 Menopause induces changes in the gingival epithelium, including atrophy, thinning, and heightened susceptibility to inflammatory changes. 5 Moreover, the significant higher proportion of postmenopausal women with plaque and calculus accumulation suggest an elevated risk of periodontal disease, where subgingival and supragingival calculus may result in gingival recession. 31

Additionally, postmenopausal women in this study reported a higher prevalence of xerostomia and burning sensation, aligning with previous research indicating these symptoms as common among postmenopausal women. 2 6 7 8 However, contradictory findings exist, as some studies report no symptoms of xerostomia in postmenopausal women despite a decrease in salivary flow rate. 9 The emergence of these symptoms may be influenced by psychological conditions, stress levels, and other local and systemic factors such as anemia, drug side effects, diabetes mellitus, and abnormal oral habits. 32 33 Xerostomia can lead to difficulties in chewing, swallowing, and speaking among the elderly, significantly reducing their oral health-related quality of life. 34

### Salivary pH and Flow Rate

Among the salivary parameters assessed in this study, only salivary flow rate demonstrated a significant difference between groups, with postmenopausal women exhibiting a significantly lower salivary flow rate. This aligns with findings from previous studies. 8 10 11 However, one study reported no significant difference in salivary flow rate between premenopausal and postmenopausal women. 12

Furthermore, despite a noticeable decrease in salivary pH among postmenopausal women, no significant difference was observed between groups. The literature presents varied results on changes in salivary pH during menopause. For instance, Mahesh et al 10 reported a statistically significant decrease in stimulated salivary pH in postmenopausal women, while Foglio-Bonda et al 13 found a statistically significant decrease in unstimulated salivary pH. Conversely, Yalçin et al 14 found no changes in salivary pH among postmenopausal women, and Prasad et al 15 reported a statistically significant higher salivary pH in this group.

The simultaneous occurrence of lower salivary pH and reduced salivary flow rate in postmenopausal women may result from salivary gland hypofunction and qualitative changes in saliva composition related to estrogen decrease during menopause. 35 This decrease in salivary pH and flow rate could also contribute to the symptoms of xerostomia observed in the postmenopausal sample in this study. 10

### Salivary Phosphate Level and Phosphorus Intake

This study found no significant difference in salivary phosphate levels between groups, contradicting some previous research indicating lower salivary phosphorus concentrations in postmenopausal women. 36 Additionally, there was no significant difference in phosphorus intake between groups, with both groups exhibiting daily phosphorus intake higher than the recommended dietary allowance (700 mg). 37 High phosphorus intakes (1,000 mg/d or higher) may increase the risk of disturbance in bone and mineral metabolism, cardiovascular disease, kidney disease, and mortality. 38 39 Regarding the oral conditions, high intake of phosphorus is associated with the increase of various inflammatory markers such interleukin-1β and C-reactive protein, potentially explaining the relatively high proportion of subjects in both groups experiencing gingivitis and dental caries. 17 18

Previous studies have indicated that serum phosphate levels correlate with age, BMI, and blood pressure. 20 40 41 However, the current study did not find any correlation between these variables and salivary phosphate levels. Additionally, salivary phosphate levels did not show a significant correlation with the daily phosphorus intake. Further investigation is recommended, taking into account additional factors that may influence salivary phosphate in oral physiological functions. These factors include other dietary components (such as calcium and protein) and salivary compositions (including sodium, potassium, calcium, magnesium, and bicarbonate), aiming to provide a comprehensive understanding of these findings.

## Conclusion

Postmenopausal women exhibited a lower salivary flow rate compared with premenopausal women, potentially contributing to a higher prevalence of gingivitis symptoms, periodontitis symptoms, caries, plaque and calculus accumulation, xerostomia, and burning sensation. However, no differences were observed in salivary pH, phosphate level, and phosphorus intake between the two groups. Additionally, salivary phosphate levels did not show a correlation with daily phosphorus intake in either group.
